# Loneliness and Social Isolation with Risk of Incident Non-alcoholic Fatty Liver Disease, UK Biobank 2006 to 2022

**DOI:** 10.34133/hds.0220

**Published:** 2025-01-07

**Authors:** Ya Miao, Xiaoke Kong, Bin Zhao, Fang Fang, Jin Chai, Jiaqi Huang

**Affiliations:** ^1^ National Clinical Research Center for Metabolic Diseases, Metabolic Syndrome Research Center, Key Laboratory of Diabetes Immunology, Ministry of Education, and Department of Metabolism and Endocrinology, The Second Xiangya Hospital of Central South University, Changsha, Hunan 410011, China.; ^2^ Xiangya School of Public Health, Central South University, Changsha, China.; ^3^ CSU-Sinocare Research Center for Nutrition and Metabolic Health, Changsha, China.; ^4^ Furong Laboratory, Changsha, China.; ^5^ Institute of Environmental Medicine, Karolinska Institutet, Stockholm, Sweden.; ^6^ Department of Gastroenterology, Institute of Digestive Diseases of PLA, Cholestatic Liver Diseases Center, and Center for Metabolic Associated Fatty Liver Disease, The First Affiliated Hospital (Southwest Hospital) to Third Military Medical University (Army Medical University), Chongqing 400038, China.

## Abstract

**Background:** Although loneliness and social isolation are proposed as important risk factors for metabolic diseases, their associations with the risk of non-alcoholic fatty liver disease (NAFLD) have not been elucidated. The aims of this study were to determine whether loneliness and social isolation are independently associated with the risk of NAFLD and to explore potential mediators for the observed associations. **Methods:** In this large prospective cohort analysis with 405,073 participants of the UK Biobank, the status of loneliness and social isolation was assessed through self-administrated questionnaires at study recruitment. The primary endpoint of interest was incident NAFLD. Multivariable-adjusted Cox proportional hazard regression models were used to calculate hazard ratios (HRs) and 95% confidence intervals for the associations between loneliness, social isolation, and risk of NAFLD. **Results:** During a median follow-up of 13.6 years, there were 5,570 cases of NAFLD identified. In the multivariable-adjusted model, loneliness and social isolation were both statistically significantly associated with an increased risk of NAFLD (HR = 1.22 and 1.13, respectively). No significant multiplicative or additive interaction was found between loneliness and social isolation on the risk of NAFLD. The mediation analysis estimated that 30.4%, 16.2%, 5.3%, 4.1%, 10.5%, and 33.2% of the loneliness–NAFLD association was mediated by unhealthy lifestyle score, obesity, current smoking, irregular physical activity, suboptimal sleep duration, and depression, respectively. On the other hand, 25.6%, 10.1%, 15.5%, 10.1%, 8.1%, 11.6%, 9.6%, 4.8%, and 3.0% of the social isolation–NAFLD association was mediated by unhealthy lifestyle score, obesity, current smoking, irregular physical activity, suboptimal sleep duration, depression, C-reactive protein, count of white blood cells, and count of neutrophils, respectively. **Conclusions:** Our study demonstrated that loneliness and social isolation were associated with an elevated risk of NAFLD, independent of other important risk factors. These associations were partially mediated by lifestyle, depression, and inflammatory factors. Our findings substantiate the importance of loneliness and social isolation in the development of NAFLD.

## Introduction

Non-alcoholic fatty liver disease (NAFLD) is one of the most prevalent chronic liver diseases, affecting approximately 30% of the global population, and is the primary precursor to cirrhosis and hepatocellular carcinoma [1]. Partially influenced by the obesity epidemic, the rising prevalence of diabetes, and aging demographics, the global incidence of NAFLD is increasing in recent years [2]. Indeed, obesity, insulin resistance, hyperlipidemia, and metabolic syndrome are well-documented risk factors for NAFLD [3]. Compelling evidence from observational studies has demonstrated that NAFLD is related to not only intrahepatic morbidity and mortality but also the risk of developing extrahepatic diseases, including cardiovascular disease (CVD) and type 2 diabetes, and overall mortality [4–6]. Identifying novel modifiable risk factors and elucidating the disease’s pathogenesis may contribute to stratifying at-risk individuals, thereby facilitating the targeted prevention of NAFLD.

Social determinants are fundamental to human welfare and play a critical role in maintaining human health. Among these determinants, loneliness and social isolation are recognized as distinct and important factors influencing health [7]. Loneliness is characterized as a distressing emotion arising from a discrepancy between desired and actual levels of social interaction [8], whereas social isolation is defined objectively as having infrequent social connections [9]. Accumulated evidence from observational studies has shown that loneliness and social isolation are associated with an increased risk of chronic diseases, including CVD, type 2 diabetes, and metabolic syndrome, as well as mortality [10–14]. However, whether loneliness and social isolation are associated with the risk of NAFLD is yet to be elucidated.

To fill these knowledge gaps, we conducted the present study to assess the associations between both loneliness and social isolation and the risk of NAFLD in the UK Biobank with more than 400,000 participants and 5,570 incident cases of NAFLD. We also assessed the additive and multiplicative interactions between loneliness and social isolation on the risk of NAFLD and explored potential mediators for the studied association.

## Materials and Methods

### Study population

The UK Biobank is a community-based prospective cohort study [15]. Between 2006 and 2010, the UK Biobank enrolled more than 500,000 participants aged 37 to 73 years from 22 assessment centers throughout England, Wales, and Scotland. During the initial assessment, all participants completed standardized questionnaires and underwent interviews conducted by nurses to collect data on demographics, lifestyle, medical history, and physical measurements. The UK Biobank received approval from the National Information Governance Board for Health and Social Care in England and Wales, the Community Health Index Advisory Group in Scotland, and the North West Multi-center Research Ethics Committee (21/NW/0157). All participants provided written informed consent at recruitment.

In the present study, we excluded participants who withdrew from the study or were pregnant at baseline (*n* = 383); participants with liver-related diseases, alcohol or drug use disorders, or cancer at/before recruitment (*n* = 65,898); and participants with missing information on loneliness or social isolation (*n* = 31,017), leaving a total of 405,073 participants in the analysis (Fig. S1). Self-reported or hospital-recorded cases of liver-related diseases and alcohol or drug use disorders were identified based on the Expert Panel Consensus Statement (Tables S1 and S2) [16]. Data on cancer at baseline were obtained through linkage to hospital inpatient records, national cancer registries, and self-reported medical conditions (Table S3).

### Assessment of exposures

The degree of loneliness was measured with the following 2 questions: (a) “Do you often feel lonely?” (“yes” = 1 point, “no” = 0 point) and (b) “How often are you able to confide in someone close to you?” (“never or almost never” = 1 point, otherwise 0 point). The loneliness score was calculated by summing up scores from these 2 questions, with a range from 0 to 2 points. Social isolation was assessed through 3 questions: (a) “Including yourself, how many people are living together in your household?” (“living alone” = 1 point, otherwise 0 point), (b) “How often do you visit friends or family or have them visit you?” (“less than once a month” = 1 point, “equal to or more than once a month” = 0 point), and (c) “Which of the following (sports club or gym, pub or social club, religious group, adult education class, other group activity) do you engage in once a week or more often?” (“none of the above” = 1 point, otherwise 0 point). The social isolation score was calculated by summing up scores from these 3 questions, with a range from 0 to 3 points. Loneliness status was pre-defined as loneliness (a score of 2 points) and no-loneliness (a score of <2 points), and social isolation status was pre-defined as isolation (a score of ≥2 points) and no-isolation (a score of <2 points) [17]. We conducted sensitivity analyses using 3-category variables for the degrees of loneliness (low degree = 0 point, moderate degree = 1 point, or high degree = 2 points) and social isolation (low degree = 0 point, moderate degree = 1 point, or high degree ≥2 points) (Table S4).

### Assessment of NAFLD

The primary endpoint of this study was the incidence of NAFLD, including non-alcoholic steatohepatitis. The diagnosis of NAFLD was identified according to the 10th revision of the International Classification of Diseases codes K76.0 and K75.8, identified through the hospital inpatient records and death registries.

### Assessment of covariates

Information on covariates was collected at baseline, including age (continuous variable), sex (men or women), ethnicity (White or others), educational level (high, intermediate, or low qualifications), Townsend deprivation index (TDI; continuous variable), body mass index (BMI; continuous variable), smoking status (never, former, or current smoker), alcohol drinking (≤14 units/week or >14 units/week), healthy diet score (<5 points or ≥5 points; details are shown in Table S5) [18], regular physical activity (yes or no), sleep duration (short: <7 h/d; normal: 7 to 8 h/d; or long: >8 h/d), and history of diabetes (yes or no), hypertension (yes or no), high cholesterol (yes or no), and CVD (yes or no). Alcohol drinking was categorized according to National Health Service recommendations [19]. Regular physical activity was defined as ≥150-min moderate activity per week, ≥75-min vigorous activity per week, an equivalent combination, moderate physical activity at least 5 d per week, or vigorous activity once per week [20]. History of diabetes, hypertension, high cholesterol, and CVD was identified according to the hospital inpatient records, “first occurrences” (category 1712), and self-reported medical conditions (details are shown in Table S3). Participants with missing values (all missing <6%) were assigned with the mode values of the study cohort for categorical variables (ethnicity, educational level, smoking status, alcohol drinking, healthy diet score, regular physical activity, and sleep duration) or the median values of the study cohort for continuous variables (TDI and BMI).

### Statistical analyses

The follow-up time was calculated from the date of recruitment to the date of NAFLD diagnosis, the date of loss to follow-up, the date of death, or the end of follow-up (2022 October 31), whichever occurred first. Cox proportional hazards models were applied to examine the hazard ratios (HRs) and their 95% confidence intervals (CIs) for the associations of loneliness and social isolation with the risk of NAFLD. The proportional hazards assumption was tested by Schoenfeld residuals, and no major violation was found. In model 1, we adjusted for age and sex. In model 2, we further adjusted for ethnicity, educational level, TDI, BMI, smoking status, alcohol drinking, healthy diet score, regular physical activity, sleep duration, and history of diabetes, hypertension, high cholesterol, and CVD, which have been shown to be associated with loneliness, social isolation, and NAFLD in previous studies [11,21]. In model 3, social isolation and loneliness were further mutually adjusted for one another. To assess the joint effect of loneliness and social isolation on NAFLD risk, we classified participants into 4 groups according to the status of loneliness and social isolation, using participants with neither loneliness nor isolation as the referent group. The additive interaction between loneliness and social isolation on risk of NAFLD was evaluated based on the relative excess risk due to interaction (RERI), attributable proportion (AP), and synergy index (SI), as well as their corresponding 95% CI [22]. For the additive interaction, a RERI of 0, an AP of 0, and an SI of 1 indicates no interaction; RERI > 0, AP > 0, and SI > 1 indicates a positive interaction; and RERI < 0, AP < 0, and SI < 1 indicates a negative interaction.

Several stratified analyses were constructed based on social isolation (yes or no, for the analysis of loneliness), loneliness (yes or no, for the analysis of social isolation), age (<60 or ≥60 years), sex (men or women), ethnicity (White or others), educational level (college or university degree or noncollege and nonuniversity degree), TDI (<median or ≥median), BMI (<30 or ≥30 kg/m^2^), current smoking (yes or no), alcohol drinking (≤14 or >14 units/week), healthy diet score (<5 or ≥5 points), regular physical activity (yes or no), normal sleep duration (yes or no), and history of chronic diseases (yes or no). A history of chronic diseases was defined as a presence of diabetes, hypertension, high cholesterol, or CVD at baseline. *P* values for multiplicative interactions were computed through likelihood ratio tests by comparing the Cox proportional hazards regression models with and without the cross-product term for each selected factor and the status of loneliness and social isolation.

Several lifestyle, psychosocial, and inflammatory factors were tested as potential mediators for the associations of loneliness and social isolation with the risk of NAFLD, including combined unhealthy lifestyle score, individual unhealthy lifestyle factors (obesity, current smoking, excess alcohol drinking, unhealthy diet, irregular physical activity, and suboptimal sleep duration), depression, C-reactive protein (CRP), count of white blood cells, and count of neutrophils. We applied the “difference method” to calculate the mediation proportion by comparing estimates from models with and without the hypothesized mediator, using the Mediate% macro in the SAS 9.4 software [23]. Data on these potential mediators were collected at baseline. Depression was defined with a score of at least 3 on the 2-item Patient Health Questionnaire [24]. Details about unhealthy lifestyle factors and the construction of unhealthy lifestyle score are shown in Table S6.

To assess the robustness of our results, we performed several sensitivity analyses: (a) To minimize bias from reverse causation, we excluded the first 2 or 5 years of follow-up [25]. (b) To decrease the influence of missingness, we excluded participants with missing data on any covariate or use multiple imputation to impute such missing data. (c) To eliminate the influence of preexisting CVD on the associations, we excluded participants with a history of CVD at baseline. (d) To alleviate the influence from depression, we further adjusted for depression in the models [24]. (e) The Fine–Gray subdistribution hazard model was utilized to examine the potential competing risk from death [26]. (f) Given the use of hospital inpatient records and death register data in the ascertainment of NAFLD, this study predominantly identified cases of NAFLD that were more severe or advanced in nature. To assess the soundness of results to such definition, we, in a sensitivity analysis, also included cases of NAFLD identified from the primary care records (category 3000) as an additional source of outcome ascertainment. (g) Given the established positive associations between the progression of NAFLD and an increased risk of liver morbidity and mortality, including cirrhotic complications, hepatocellular carcinoma, and liver-related mortality [6], we used severe liver diseases as a secondary outcome in another sensitivity analysis, including cirrhosis, hepatocellular carcinoma, liver failure, and liver-related mortality (Table S1).

All statistical analyses were conducted using SAS version 9.4 (SAS Institute Inc.) and R version 4.3.1 (R Development Core Team), and 2-sided statistical tests were performed. In the primary analysis, a *P* value of <0.05 was considered statistically significant. To account for multiple testing, we used Bonferroni correction to define the statistical significance: 0.05/13 = 0.0038 (13 tests) for the interaction tests in stratified analyses.

## Results

The baseline characteristics of the 405,073 participants according to the status of loneliness and social isolation are presented in Table 1. The mean (SD) age of the participants was 56.2 (8.1) years, and 46.5% of the participants were men. A total of 19,033 (4.7%) participants were defined as having loneliness, and 35,941 (8.9%) participants were defined as having social isolation. Participants with loneliness or social isolation were more likely to be men, non-White, and current smokers and had a lower socioeconomic status and a higher BMI, compared to others. They were also more likely to have a history of diabetes, hypertension, high cholesterol, CVD, or depression and were less likely to have a college or a university degree, a regular physical activity, or a normal sleep duration (7 to 8 h). Participants with social isolation were more likely to have a healthy diet, whereas those with loneliness were less likely to have a healthy diet.

**Table 1.** Baseline characteristics of the participants in the study. Values are means (SD) or percentages. Continuous variables are presented as mean ± SD, and categorical variables are presented as percentages.

Characteristics	All	Loneliness		Social isolation	
		No	Yes	No	Yes
Number of participants	405,073	386,040	19,033	369,132	35,941
Age (years), mean (SD)	56.2 (8.1)	56.2 (8.1)	55.7 (7.9)	56.2 (8.1)	56.3 (7.8)
Men (%)	46.5	46.3	51.7	46.2	49.5
White (%)	95.1	95.2	93.7	95.3	93.2
Educational level (%) ^a^					
High qualifications	33.4	33.8	24.2	33.6	30.6
Intermediate qualifications	50.6	50.5	52.7	51.0	47.2
Low qualifications	16.0	15.7	23.1	15.4	22.2
Townsend deprivation index, mean (SD)	−1.4 (3.0)	−1.4 (3.0)	−0.6 (3.4)	−1.5 (3.0)	−0.2 (3.4)
BMI (kg/m^2^), mean (SD)	27.4 (4.8)	27.4 (4.7)	28.5 (5.5)	27.4 (4.7)	27.9 (5.4)
Smoking status (%)					
Never smoker	55.8	56.1	49.8	56.4	49.8
Previous smoker	34.2	34.2	33.9	34.4	32.7
Current smoker	10.0	9.7	16.3	9.3	17.5
Alcohol drinking >14 units/week (%)	33.2	33.3	30.1	34.1	23.9
Healthy diet (%) ^b^	15.1	15.1	14.1	14.9	17.0
Regular physical activity (%)	78.4	78.7	72.2	79.4	67.9
Sleep duration (%)					
Normal, 7–8 h/d	68.4	69.1	54.5	69.3	60.1
Short, <7 h/d	24.2	23.6	37.2	23.5	31.5
Long, >8 h/d	7.3	7.3	8.3	7.2	8.5
Diabetes (%)	5.2	5.0	8.7	4.9	7.8
Hypertension (%)	29.0	28.7	34.8	28.6	33.5
High cholesterol (%)	18.4	18.2	23.1	18.1	21.7
CVD (%)	6.6	6.4	10.4	6.4	8.6
Depression (%)	5.4	4.5	22.8	4.8	11.2

BMI, body mass index; CSE, Certificate of Secondary Education; CVD, cardiovascular disease; GCSE, General Certificate of Secondary Education; HNC, Higher National Certificate; HND, Higher National Diploma; NVQ, National Vocational Qualification; SD, standard deviation.

^a^ High qualifications (college or university degree), intermediate qualifications (A levels/AS levels or equivalent, O levels/GCSEs or equivalent, CSEs or equivalent, NVQ or HND or HNC or equivalent, or other professional qualifications), low qualifications (none of the above).

^b^ Healthy diet: healthy diet score ≥ 5.

During a median follow-up of 13.6 years (5,393,437 person-years in total), we identified 5,570 incident cases of NAFLD (Table 2). In the sex- and age-adjusted model (model 1), loneliness and social isolation were associated with a higher risk of NAFLD (loneliness: HR = 1.80, 95% CI: 1.64, 1.99; social isolation: HR = 1.53, 95% CI: 1.41, 1.65) (Table 2). In model 2, the associations attenuated but remained statistically significant. Compared with participants without loneliness or social isolation, those with loneliness or social isolation had a 24% and a 15% elevated risk of NAFLD, respectively. In model 3, after mutual adjustment for social isolation and loneliness, the associations remained unchanged (loneliness: HR = 1.22, 95% CI: 1.11, 1.35; social isolation: HR = 1.13, 95% CI: 1.04, 1.23). Similar results were observed when we used the 3-category variables for degrees of loneliness and social isolation. In model 3, compared to a low degree of loneliness (0 point) or social isolation (0 point), the HRs (95% CIs) for risk of NAFLD were 1.19 (1.12, 1.26) and 1.29 (1.17, 1.43) for a moderate or high degree of loneliness and were 1.13 (1.07, 1.20) and 1.18 (1.08, 1.29) for a moderate or high degree of social isolation, respectively (all *P* trend <0.001, Table S7). When analyzing the joint effect of loneliness and social isolation, the HRs were 1.15 (95% CI: 1.06, 1.26) for social isolation alone, 1.26 (95% CI: 1.13, 1.42) for loneliness alone, and 1.28 (95% CI: 1.07, 1.54) for both loneliness and social isolation, using neither loneliness nor social isolation as the reference (*P* for trend <0.001). We did not observe a significant additive interaction between loneliness and social isolation on the risk of NAFLD, as the RERI was −0.14 (−0.42, 0.14), the AP was −0.11 (−0.35, 0.13), and the SI was 0.66 (0.26, 1.68) (Table S8).

**Table 2.** Associations of loneliness and social Isolation with the risk of incident NAFLD

Models	Loneliness		*P* value	Social isolation		*P* value
	No	Yes		No	Yes	
No. of cases/total	5,123/386,040	447/19,033		4,869/369,132	701/35,941	
Model 1, ^a^ HR (95% CI)	1 (reference)	1.80 (1.64, 1.99)	<0.001	1 (reference)	1.53 (1.41, 1.65)	<0.001
Model 2, ^b^ HR (95% CI)	1 (reference)	1.24 (1.13, 1.37)	<0.001	1 (reference)	1.15 (1.06, 1.25)	<0.001
Model 3, ^c^ HR (95% CI)	1 (reference)	1.22 (1.11, 1.35)	<0.001	1 (reference)	1.13 (1.04, 1.23)	0.003

CI, confidence interval; CVD, cardiovascular disease; HR, hazard ratio; NAFLD, non-alcoholic fatty liver disease.

^a^ Model 1: adjusted for age and sex.

^b^ Model 2: adjusted for age, sex, ethnicity, educational level, Townsend deprivation index, body mass index, smoking status, alcohol drinking, healthy diet score, regular physical activity, sleep duration, and history of diabetes, hypertension, high cholesterol, and CVD at baseline.

^c^ Model 3: all covariables adjusted for in model 2 together with mutual adjustment of social isolation and loneliness for one another.

In the stratified analyses using model 3, we observed similar estimates for the loneliness–NAFLD or social isolation–NAFLD association across subgroups of age, sex, ethnicity, educational level, TDI, BMI, current smoking, alcohol drinking, healthy diet score, regular physical activity, and normal sleep duration (all *P* for interaction >0.0038, Figure). No multiplicative interaction was observed between loneliness and social isolation for the risk of NAFLD (*P* for interaction = 0.26, Figure). The social isolation–NAFLD association was, however, statistically significantly modified by the history of chronic diseases (*P* for interaction = 0.0002), with a stronger association noted among participants without a history of chronic diseases at recruitment (HR = 1.35, 95% CI: 1.19, 1.53). We also observed a stronger association between social isolation and NAFLD among participants at younger ages (<60 years at recruitment: HR = 1.22, 95% CI: 1.10, 1.35) and with alcohol drinking >14 units/week (HR = 1.29, 95% CI: 1.10, 1.51). The tests for interaction did not reach statistical significance after correction for multiple testing (*P* for interaction = 0.01 and 0.04, respectively, Figure).

**Figure.**
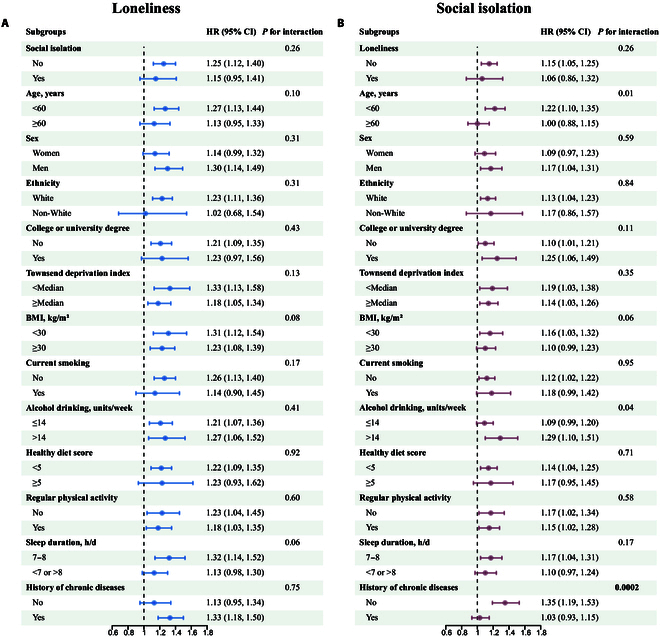
(A) Stratified analyses of the association between loneliness and NAFLD risk. Adjusted for age; sex; ethnicity; educational level; Townsend deprivation index; body mass index; smoking status; alcohol drinking; healthy diet score; regular physical activity; sleep duration; history of diabetes, hypertension, high cholesterol, and CVD at baseline; and social isolation. (B) Stratified analyses of the association between social isolation and NAFLD risk. Adjusted for age; sex; ethnicity; educational level; Townsend deprivation index; body mass index; smoking status; alcohol drinking; healthy diet score; regular physical activity; sleep duration; history of diabetes, hypertension, high cholesterol, and CVD at baseline; and loneliness. BMI, body mass index; CI, confidence interval; CVD, cardiovascular disease; HR, hazard ratio; NAFLD, non-alcoholic fatty liver disease.

Results from mediation analyses showed that 30.4% (95% CI: 20.5%, 42.6%), 16.2% (95% CI: 10.7%, 24.0%), 5.3% (95% CI: 2.9%, 9.5%), 4.1% (95% CI: 1.8%, 9.0%), 10.5% (95% CI: 5.9%, 18.2%), and 33.2% (95% CI: 16.7%, 55.1%) of the loneliness–NAFLD association were statistically significantly accounted by unhealthy lifestyle score, obesity, current smoking, irregular physical activity, suboptimal sleep duration, and depression, respectively (Table 3). Additionally, 25.6% (95% CI: 16.2%, 38.1%), 10.1% (95% CI: 4.6%, 20.9%), 15.5% (95% CI: 7.9%, 28.2%), 10.1% (95% CI: 4.7%, 20.5%), 8.1% (95% CI: 3.9%, 16.0%), 11.6% (95% CI: 5.0%, 24.8%), 9.6% (95% CI: 3.7%, 22.5%), 4.8% (95% CI: 2.0%, 11.2%), and 3.0% (95% CI: 1.1%, 8.1%) of the social isolation–NAFLD association were statistically significantly mediated through unhealthy lifestyle score, obesity, current smoking, irregular physical activity, suboptimal sleep duration, depression, CRP, count of white blood cells, and count of neutrophils, respectively (Table 3).

**Table 3.** Mediating effect of potential mediators in the associations of loneliness and social isolation with the risk of NAFLD. Adjusted for age, sex, ethnicity, educational level, Townsend deprivation index, body mass index, smoking status, alcohol drinking, healthy diet score, regular physical activity, sleep duration, and history of diabetes, hypertension, high cholesterol, and CVD at baseline and mutually adjusted for social isolation and loneliness.

Mediating factors	HR (95% CI)		Mediation proportion (%) (95% CI)	*P* value for mediation
	Unadjusted for the factor	Adjusted for the factor		
Loneliness				
Unhealthy lifestyle score	1.37 (1.23, 1.52)	1.24 (1.11, 1.38)	30.4 (20.5, 42.6)	<0.001
Obesity	1.32 (1.19, 1.46)	1.26 (1.14, 1.39)	16.2 (10.7, 24.0)	<0.001
Current smoking	1.24 (1.13, 1.37)	1.23 (1.11, 1.36)	5.3 (2.9, 9.5)	<0.001
Excess alcohol drinking ^a^	1.22 (1.11, 1.35)	1.22 (1.11, 1.35)	None	ND
Unhealthy diet ^b^	1.22 (1.10, 1.34)	1.22 (1.10, 1.34)	None	ND
Irregular physical activity ^c^	1.20 (1.08, 1.33)	1.19 (1.07, 1.32)	4.1 (1.8, 9.0)	<0.001
Suboptimal sleep duration ^d^	1.24 (1.12, 1.37)	1.21 (1.10, 1.34)	10.5 (5.9, 18.2)	<0.001
Depression	1.24 (1.12, 1.38)	1.16 (1.04, 1.29)	33.2 (16.7, 55.1)	<0.001
C-reactive protein (mg/l) ^e^	1.26 (1.14, 1.40)	1.26 (1.14, 1.39)	None	ND
White blood cells (10^9^ cells/l) ^e^	1.23 (1.11, 1.36)	1.23 (1.11, 1.36)	None	ND
Neutrophils (10^9^ cells/l) ^e^	1.23 (1.11, 1.36)	1.22 (1.11, 1.35)	None	ND
Social isolation				
Unhealthy lifestyle score	1.24 (1.14, 1.35)	1.17 (1.07, 1.28)	25.6 (16.2, 38.1)	<0.001
Obesity	1.15 (1.05, 1.24)	1.13 (1.04, 1.23)	10.1 (4.6, 20.9)	<0.001
Current smoking	1.16 (1.07, 1.25)	1.13 (1.04, 1.23)	15.5 (7.9, 28.2)	<0.001
Excess alcohol drinking ^a^	1.13 (1.04, 1.22)	1.13 (1.04, 1.23)	None	ND
Unhealthy diet ^b^	1.14 (1.05, 1.24)	1.14 (1.05, 1.24)	None	ND
Irregular physical activity ^c^	1.17 (1.07, 1.28)	1.15 (1.06, 1.26)	10.1 (4.7, 20.5)	<0.001
Suboptimal sleep duration ^d^	1.14 (1.05, 1.24)	1.13 (1.04, 1.23)	8.1 (3.9, 16.0)	<0.001
Depression	1.13 (1.04, 1.23)	1.11 (1.02, 1.21)	11.6 (5.0, 24.8)	<0.001
C-reactive protein (mg/l) ^e^	1.12 (1.03, 1.22)	1.11 (1.02, 1.21)	9.6 (3.7, 22.5)	<0.001
White blood cells (10^9^ cells/l) ^e^	1.12 (1.03, 1.22)	1.12 (1.03, 1.21)	4.8 (2.0, 11.2)	<0.001
Neutrophils (10^9^ cells/l) ^e^	1.12 (1.03, 1.22)	1.12 (1.03, 1.21)	3.0 (1.1, 8.1)	0.002

CI, confidence interval; CVD, cardiovascular disease; HR, hazard ratio; NAFLD, non-alcoholic fatty liver disease; ND, no data.

^a^ Excess alcohol drinking: >14 units/week.

^b^ Unhealthy diet: healthy diet score < 5.

^c^ Irregular physical activity: failing to meet the criterion of ≥150-min moderate activity per week or ≥75-min vigorous activity per week or equivalent combination or moderate physical activity at least 5 d a week or vigorous activity once a week.

^d^ Suboptimal sleep duration: <7 or >8 h/d.

^e^ Levels of inflammatory biomarkers were natural log-transformed before analyses.

Our results did not appreciably change after excluding the first 2 years (loneliness: HR = 1.23, 95% CI: 1.11, 1.36; social isolation: HR = 1.14, 95% CI: 1.04, 1.23) or 5 years of follow-up (loneliness: HR = 1.18, 95% CI: 1.05, 1.31; social isolation: HR = 1.16, 95% CI: 1.06, 1.27), excluding participants with missing value on covariates (loneliness: HR = 1.18, 95% CI: 1.06, 1.32; social isolation: HR = 1.14, 95% CI: 1.05, 1.25), excluding participants with a history of CVD at baseline (loneliness: HR = 1.16, 95% CI: 1.04, 1.30; social isolation: HR = 1.14, 95% CI: 1.04, 1.24), further adjusting for depression (loneliness: HR = 1.15, 95% CI: 1.04, 1.27; social isolation: HR = 1.12, 95% CI: 1.03, 1.21), addressing competing risk from death (loneliness: HR = 1.22, 95% CI: 1.10, 1.34; social isolation: HR = 1.10, 95% CI: 1.02, 1.20), or using an multiple-imputation approach (loneliness: HR = 1.22, 95% CI: 1.10, 1.35; social isolation: HR = 1.13, 95% CI: 1.04, 1.22, Table S9). Our primary findings remained unchanged when including the primary care records as an additional source of outcome ascertainment (loneliness: HR = 1.16, 95% CI: 1.06, 1.27; social isolation: HR = 1.11, 95% CI: 1.03, 1.20) or using severe liver diseases as a secondary outcome (loneliness: HR = 1.18, 95% CI: 1.05, 1.33; social isolation: HR = 1.14, 95% CI: 1.04, 1.25, Table S9).

## Discussion

In this large prospective cohort study of the UK Biobank with more than 400,000 participants, we found that loneliness and social isolation were independently associated with a higher risk of NAFLD, with participants who had a status of loneliness or social isolation experiencing a 22% and a 13% increased disease risk, respectively. The positive associations remained generally similar across a series of stratified and sensitivity analyses, demonstrating the robustness of our results. Our findings also elucidated that the loneliness–NAFLD and social isolation–NAFLD associations were partially mediated through obesity, current smoking, irregular physical activity, suboptimal sleep duration, and depression, as well as inflammatory biomarkers like CRP and immune cell counts.

Although previous studies have shown a high correlation between depression and NAFLD [27,28], data on the roles of psychosocial factors in the pathogenesis of NAFLD are still scarce. To the best of our knowledge, this is the first study to examine the associations between loneliness and social isolation and the risk of NAFLD. Regardless, accumulating evidence from previous observational studies has shown that loneliness and social isolation are important risk factors for different chronic diseases, including type 2 diabetes [11], metabolic syndrome [12,29], and CVD [10,30,31], as well as mortality [13] among individuals with metabolic diseases or the general population. In a recent study including 423,503 adults from the UK Biobank and 13,800 adults from the China Health and Retirement Longitudinal Study, loneliness and social isolation were both found to be associated with an elevated risk of type 2 diabetes, regardless of genetic predisposition [11]. The Nord-Trøndelag Health Study with 26,990 participants and 10 years of follow-up demonstrated a positive association between loneliness and the risk of metabolic syndrome and showed that the association was partially mediated through depressive symptoms [32]. In a recent meta-analysis involving 90 prospective cohort studies with more than 2 million individuals, loneliness and social isolation were shown to be associated with a 14% and a 32% increased risk of overall mortality, respectively [13].

Several biological mechanisms might explain our findings. Loneliness and social isolation can trigger pathophysiological changes, including the dysregulation of the hypothalamic–pituitary–adrenal axis, elevated pro-inflammatory and immune responses, increased oxidative stress, disruption of parasympathetic function, and impairment of energy metabolism [33–35]. For example, previous studies have demonstrated that individuals experiencing loneliness have higher levels of morning cortisol release, suggesting an exacerbated activation of the hypothalamic–pituitary–adrenal axis [36]. In addition, findings based on the Whitehall II cohort of healthy middle-aged individuals showed that individuals with social isolation had an increased cortisol arousal response and a higher total cortisol release [37].

Suboptimal lifestyle factors are more prevalent among individuals experiencing loneliness or social isolation, including obesity [38–40], smoking [41,42], physical inactivity [42–44], and poor sleep quality [45,46]. Accumulating evidence from observational studies has demonstrated that unhealthy lifestyle factors may escalate oxidative stress [47], promote systemic chronic inflammation [48], and exacerbate insulin resistance [49]. These pathways have all been known as pivotal to the pathogenesis of NAFLD [50–53]. In contrast, adherence to a favorable lifestyle could prevent 66.8% (95% CI: 58.5%, 75.1%) of severe NAFLD [21]. Our findings showed that unhealthy lifestyle score accounted for 30.4% and 25.6% of the associations of loneliness and social isolation with the risk of NAFLD, respectively. Our findings provide evidence to support that interventions targeting modifiable lifestyle factors may contribute to the prevention of NAFLD, particularly among individuals experiencing loneliness or social isolation.

Of note, loneliness and social isolation represent different aspects of social connections [54]. In the present study, we found that the loneliness–NAFLD association was stronger than the social isolation–NAFLD association, suggesting that the deleterious role of loneliness might be stronger than that of social isolation. In addition, neither additive nor multiplicative interaction was detected between loneliness and social isolation on NAFLD risk. One plausible interpretation is that, in contrast to the subjective experience of loneliness with negative emotions, social isolation may not provoke adverse emotional responses in all individuals, particularly those who exhibit a preference of solitude [55]. However, with the experience of higher emotional vulnerability, coupled with hypervigilance, and perseverative cognition, loneliness appears to exert a considerable impact on psychiatric health, including stress reaction and depressive symptoms, and may contribute to the development and progression of metabolic diseases [32,56]. For example, observational studies have shown a positive association between depression and the risk of NAFLD [27,28], and a recent Mendelian randomization study demonstrated that genetic susceptibility to depression was associated with the risk of developing NAFLD [57]. In the present study, our findings demonstrated that depression accounted for 33.2% of the loneliness–NAFLD association, greater than the 11.6% mediation noted in the association between social isolation and risk of NAFLD. Thus, in addition to mitigating the public health concern of social isolation, it is also crucial to incorporate psychological interventions to alleviate individual feelings of loneliness in the efforts of preventing NAFLD.

The primary strengths of the present study include the prospective design and data collection, the large sample size, complete follow-up, and detailed information on lifestyles and disease-associated factors, which enabled us to control for multiple important covariates and conduct comprehensive stratified and sensitivity analyses. Several limitations of the study should also be considered. Firstly, given the observational nature of the study design, our findings cannot directly evidence a causal link between loneliness, social isolation, and the risk of NAFLD. Secondly, loneliness and social isolation were assessed only once at baseline and may not capture all social connections of the participants. However, the scaling approach has been established in previous studies using different cohorts, reflecting its application and efficacy in large population-based studies [17,30,31]. Thirdly, as the majority of participants in the UK Biobank study are white European, whether our findings can be generalized to diverse ethnic populations warrants further exploration. Fourthly, given the use of hospital inpatient care and death data, the present study predominantly focused on more severe or advanced NAFLD cases. To assess the soundness of the results to such focus, we also included NAFLD ascertained through primary care records as an additional source of outcome ascertainment in a sensitivity analysis, rendering, however, essentially unchanged results.

In conclusion, in this large prospective cohort of more than 400,000 participants in the UK Biobank, we found that loneliness and social isolation were associated with an increased risk of NAFLD, independent of several important risk factors. The associations were partially mediated through obesity, smoking, irregular physical activity, suboptimal sleep duration, and depression, as well as inflammatory factors. Our findings substantiate the importance of loneliness and social isolation in the prevention of NAFLD. Intervention strategies targeted at alleviation concerns from these factors warrants further investigation.

## Ethical Approval

The UK Biobank received approval from the National Information Governance Board for Health and Social Care in England and Wales, the Community Health Index Advisory Group in Scotland, and the North West Multi-center Research Ethics Committee (21/NW/0157). All participants provided written informed consent at recruitment.

## Data Availability

Data from UK Biobank are available to all researchers upon submitting an application. This research was performed using the UK Biobank Resource under Application ID 98583.
