# Supplementary material for: Loneliness and Social Isolation with Risk of Incident Non-alcoholic Fatty Liver Disease, UK Biobank 2006 to 2022
Source: Health Data Sci. 2025 Jan 7;5:0220. doi: 10.34133/hds.0220 (PMC11704091; doi:10.34133/hds.0220)

**Supplemental Table 1.** Identification of Liver-Related Diseases and Alcohol/Drug Use Disorders based on the Hospital Inpatient Records in the UK Biobank ^a^

| **Diagnosis** | **ICD-9** | **ICD-10** |
| --- | --- | --- |
| **Liver-related diseases** | | |
| NAFLD, all | 571.8 | K76.0 |
| NASH | --- | K75.8 |
| ALD | 571.0-571.3 | K70 |
| Viral hepatitis | 070 | B16, B17, B18, B19 |
| Autoimmune liver disease (AIH, PBC, PSC) | 571.6, 576.1 | K83.0A, K83.0F K74.3, K75.4 |
| Hemochromatosis | 275.0 | E83.1 |
| Wilson’s disease | 275.1 | E83.0B |
| Alpha-1-antirypsin deficiency | 277.6 | E88.0A, E88.0B |
| Budd-Chiari syndrome | 453.0 | I82.0, K76.5 |
| Chronic hepatitis, unspecified | 571.4 | K73.9, K73.2 |
| Secondary or unspecified biliary cirrhosis | 571.6 | K74.4, K74.5 |
| Compensated cirrhosis | 571.5, 456.1, 456.21 | K74.6, I85.9, I98.2, I86.4 |
| Decompensated cirrhosis | 456.0, 456.20, 789.5, 572.2, 572.4, 572.3 | I85.0, I98.3, R18, K76.7, K76.6 |
| Liver transplantation | V42.7 | Z94.4 |
| Liver cancer outcomes | I55.0, I55.2 | C22.0, C22.9 |
| Liver failure | 572.8, 570 | K72.1, K72.9, K72.0 |
| Portal vein thrombosis | --- | I81.9, K75.1 |
| Hepatic fibrosis or sclerosis or fibrosis with sclerosis | --- | K74.0, K74.1, K74.2 |
| **Alcohol/drug use disorders** | | |
| Codes associated with alcohol use disorders | 303, 305.0 | F10 |
| Codes associated with somatic consequence of alcohol (except ALD) | 291, 357.5, 425.5, 535.3, 980.1, 980.9 | E24.4, G62.1, I42.6, K29.2, G31.2, G72.1, K85.2, K86.0, T51.0, T51.9, Y57.3, X65, Z50.2, Z71.4, Z72.1 |
| Codes associated with drug use disorders except nicotine/caffeine | 305.1-305.9 | F11-F14, F16, F18, F19 |

Abbreviations: AIH=autoimmune hepatitis; ALD=alcohol-associated liver disease; ICD-9=the 9^th^ revision of the International Classification of Diseases; ICD-10=the 10^th^ revision of the International Classification of Diseases; NAFLD=non-alcoholic fatty liver disease; NASH=non-alcoholic steatohepatitis; PBC=primary biliary cholangitis; PSC=primary sclerosing cholangitis

^a^ A composite definition of severe liver diseases (ICD-10 codes: I85.9, I86.4, I98.2, K74.1, K74.2, K74.6, I85.0, I98.3, K76.6, K76.7, R18, C22.0, K72.0, K72.1, K72.9) was used as a secondary outcome in the sensitivity analysis.

**Supplemental Table 2.** Identification of Liver-Related Diseases and Alcohol/Drug Use Disorders Based on the Self-Reported Data in the UK Biobank

| **Self-reported (data field 20002)** | | | |
| --- | --- | --- | --- |
| **Code** | **Description** | **Code** | **Description** |
| 1141 | Oesophageal varicies | 1496 | Alpha-1 antitrypsin deficiency |
| 1155 | Hepatitis | 1506 | Primary biliary cirrhosis |
| 1156 | Infective/viral hepatitis | 1507 | Haemochromatosis |
| 1157 | Non-infective hepatitis | 1579 | Hepatitis b |
| 1158 | Liver failure/cirrhosis | 1580 | Hepatitis c |
| 1408 | Alcohol dependency | 1581 | Hepatitis d |
| 1409 | Opioid dependency | 1582 | Hepatitis e |
| 1475 | Sclerosing cholangitis | 1604 | Alcoholic liver disease / alcoholic cirrhosis |
| **Self-reported (data field 20001)** | | | |
| **Code** | **Description** | **Code** | **Description** |
| 1024 | Liver/hepatocellular cancer | --- | --- |

**Supplemental Table 3.** Field IDs and Codes Used to Identify Chronic Diseases at Baseline in the UK Biobank

|  | **Hospital inpatient records (ICD-9)** | **Hospital inpatient records (ICD-10)** | **Cancer register** | **First occurrences** | **Self-reported fields** |
| --- | --- | --- | --- | --- | --- |
| **Cancer at baseline** | 41271 (140-209), 41281 | 41270 (C00-C97), 41280 | 40005 | **/** | 2453, 20001 |
| **Diabetes at baseline** | 41271 (250), 41281 | 41270 (E10-E14), 41280 | / | 130706, 130708, 130710, 130712, 130714 | 2443, 2976, 6153, 6177, 20002, 20003 |
| **Hypertension at baseline** | 41271 (401-405), 41281 | 41270 (I10-I13, I15), 41280 | / | 131286, 131288, 131290, 131292, 131294 | 2966, 6177, 6153, 6150, 20002 |
| **High cholesterol at baseline** | 41271 (2720), 41281 | 41270 (E780), 41280 | / | 130814 | 6153, 6177, 20002 |
| **CVD at baseline** | 41271 (410-414, 428-431, 434,  436), 41281 | 41270 (I20-I25, I50, I60, I61, I63, I64), 41280 | / | 131296, 131298, 131300, 131302, 131304, 131306, 131354, 131360, 131362, 131366, 131368 | 6150, 3894, 3627, 4056, 20002 |

Abbreviations: CVD=cardiovascular disease; ICD-9=the 9^th^ revision of the International Classification of Diseases; ICD-10=the 10^th^ revision of the International Classification of Diseases

**Supplemental Table 4.** Detailed Information on the Scales of Loneliness and Social Isolation in the UK Biobank

| **Exposures** | **Field ID** | **Description** | **Questions** |
| --- | --- | --- | --- |
| Loneliness | 2020 | Feeling lonely | “Do you often feel lonely?” |
|  | 2110 | Able to confide | “How often are you able to confide in someone close to you?” |
| Social isolation | 709 | Number in household | “Including yourself, how many people are living together in your household?” |
|  | 1031 | Frequency of friend/family visits | “How often do you visit friends or family or have them visit you?” |
|  | 6160 | Leisure/social activities | “Which of the following leisure/social activities do you engage in once a week or more often?” |

**Supplemental Table 5.** Dietary Components and Criteria for Dietary Score ^a^

| **Dietary components** | **Intake goals** | **Score** |
| --- | --- | --- |
| Fruit | ≥3 servings/day | 1 |
| Vegetable | ≥3 servings/day | 1 |
| Whole grains | ≥3 servings/day | 1 |
| (Shell)fish | ≥2 servings/week | 1 |
| Dairy | ≥2 servings/day | 1 |
| Vegetable oils | ≥2 servings/day | 1 |
| Refined grains | ≤2 servings/day | 1 |
| Processed meats | ≤1 serving/week | 1 |
| Unprocessed meats | ≤2 servings/week | 1 |
| Sugar-sweetened beverages | No consumption | 1 |

^a^ The dietary score was calculated by summing up all scores from the dietary components. A healthy diet was defined by a dietary score of 5 or above.

**Supplemental Table 6.** Definition of Unhealthy Lifestyle Factors and the Construction of Unhealthy Lifestyle Score ^a^

| **Unhealthy Lifestyle factors** | **Definition** |
| --- | --- |
| Obesity | Body mass index ≥30 kg/m^2^ |
| Current smoking | Current smoker |
| Excess alcohol drinking | Alcohol drinking >14 units/week |
| Unhealthy diet | Healthy diet score <5 |
| Irregular physical activity | Regular physical activity was defined as ≥150 minutes moderate activity per week, or ≥75 minutes vigorous activity per week, or equivalent combination, or moderate physical activity at least 5 days a week, or vigorous activity once a week, otherwise irregular. |
| Suboptimal sleep duration | <7 hours/day or >8 hours/day |

^a^ Unhealthy lifestyle score was generated through body mass index (score of 1: ≥30 kg/m^2^), smoking (score of 1: current), alcohol drinking (score of 1: >14 units/week), healthy diet score (score of 1: <5), physical activity (score of 1: irregular) and sleep duration (score of 1: <7 hours/day or >8 hours/day)

**Supplemental Table 7.** Associations of Loneliness and Social Isolation with Risk of NAFLD

| Models | **Loneliness scale** | | | |
| --- | --- | --- | --- | --- |
|  | **Low degree (0 point)** | **Moderate degree (1 point)** | **High degree (2 points)** | ***P*-trend** |
| No. of cases/total | 3,460/291,381 | 1,663/94,659 | 447/19,033 | **---** |
| Model 1^a^, HR (95% CI) | 1 (Reference) | 1.50 (1.41, 1.59) | 2.02 (1.83, 2.23) | < 0.001 |
| Model 2^b^, HR (95% CI) | 1 (Reference) | 1.21 (1.14, 1.29) | 1.33 (1.20, 1.47) | < 0.001 |
| Model 3^c^, HR (95% CI) | 1 (Reference) | 1.19 (1.12, 1.26) | 1.29 (1.17, 1.43) | < 0.001 |
| Models | **Social isolation scale** | | | |
|  | **Low degree (0 point)** | **Medium degree (1 point)** | **High degree (≥2 points)** | ***P*-trend** |
| No. of cases/total | 2,498/215,360 | 2,371/153,772 | 701/35,941 | **---** |
| Model 1^a^, HR (95% CI) | 1 (Reference) | 1.35 (1.28, 1.43) | 1.75 (1.61, 1.90) | < 0.001 |
| Model 2^b^, HR (95% CI) | 1 (Reference) | 1.15 (1.09, 1.22) | 1.24 (1.14, 1.35) | < 0.001 |
| Model 3^c^, HR (95% CI) | 1 (Reference) | 1.13 (1.07, 1.20) | 1.18 (1.08, 1.29) | < 0.001 |

Abbreviations: CI=confidence interval; CVD=cardiovascular disease; HR=hazard ratio; NAFLD=non-alcoholic fatty liver disease

^a^ Model 1: adjusted for age and sex;

^b^ Model 2: adjusted for age, sex, ethnicity, educational level, Townsend deprivation index, body mass index, smoking status, alcohol drinking, healthy diet score, regular physical activity, sleep duration, and history of diabetes, hypertension, high cholesterol, and CVD at baseline;

^c^ Model 3: all covariables adjusted for in Model 2 together with mutual adjustment of loneliness and social isolation for one another.**Supplemental Table 8.** Joint Effect of Loneliness and Social Isolation on the Risk of NAFLD ^a^

| **Social isolation** | **Loneliness, HR (95% CI)** | | ***P* for trend** |
| --- | --- | --- | --- |
|  | No | Yes |  |
| **No** | 1 (Reference) | 1.26 (1.13, 1.42) | <0.001 |
| **Yes** | 1.15 (1.06, 1.26) | 1.28 (1.07, 1.54) |  |
| **RERI (95% CI)** | -0.14 (-0.42, 0.14) | |  |
| **AP (95% CI)** | -0.11 (-0.35, 0.13) | |  |
| **SI (95% CI)** | 0.66 (0.26, 1.68) | |  |

Abbreviations: AP=attributable proportion; CI=confidence interval; CVD=cardiovascular disease; HR=hazard ratio; NAFLD=non-alcoholic fatty liver disease; RERI=relative excess risk due to interaction; SI=synergy index

^a^ Models were adjusted for age, sex, ethnicity, educational level, Townsend deprivation index, body mass index, smoking status, alcohol drinking, healthy diet score, regular physical activity, sleep duration, and history of diabetes, hypertension, high cholesterol, and CVD at baseline.

**Supplemental Table 9**. Sensitivity Analyses on the Associations of Loneliness and Social Isolation with Risk of NAFLD ^a^

| **Sensitivity analysis** | **Loneliness, HR (95% CI)** | |  | **Social isolation, HR (95% CI)** | |
| --- | --- | --- | --- | --- | --- |
|  | No | Yes |  | No | Yes |
| Excluding first 2 years of follow-up | 1 (Reference) | 1.23 (1.11, 1.36) |  | 1 (Reference) | 1.14 (1.04, 1.23) |
| Excluding first 5 years of follow-up | 1 (Reference) | 1.18 (1.05, 1.31) |  | 1 (Reference) | 1.16 (1.06, 1.27) |
| Excluding participants with missing data on covariates | 1 (Reference) | 1.18 (1.06, 1.32) |  | 1 (Reference) | 1.14 (1.05, 1.25) |
| Excluding participants with a history of CVD | 1 (Reference) | 1.16 (1.04, 1.30) |  | 1 (Reference) | 1.14 (1.04, 1.24) |
| Further adjusted for depression ^b^ | 1 (Reference) | 1.15 (1.04, 1.27) |  | 1 (Reference) | 1.12 (1.03, 1.21) |
| Competing risk model | 1 (Reference) | 1.22 (1.10, 1.34) |  | 1 (Reference) | 1.10 (1.02, 1.20) |
| Multiple imputation approach ^c^ | 1 (Reference) | 1.22 (1.10, 1.35) |  | 1 (Reference) | 1.13 (1.04, 1.22) |
| Incident NAFLD (identified from hospital, death and primary care records) | 1 (Reference) | 1.16 (1.06, 1.27) |  | 1 (Reference) | 1.11 (1.03, 1.20) |
| Severe liver diseases as an outcome | 1 (Reference) | 1.18 (1.05 1.33) |  | 1 (Reference) | 1.14 (1.04, 1.25) |

Abbreviations: CI=confidence interval; CVD=cardiovascular disease; HR=hazard ratio; NAFLD=non-alcoholic fatty liver disease

^a^ Models were adjusted for age, sex, ethnicity, educational level, Townsend deprivation index, body mass index, smoking status, alcohol drinking, healthy diet score, regular physical activity, sleep duration, and history of diabetes, hypertension, high cholesterol, and CVD at baseline, and mutually adjusted for social isolation and loneliness;

^b^ Participants with missing values for depression was assigned with the mode values of the study cohort.

^c^ Multiple imputation (n = 5) was used to impute missing values of covariates (SAS program, PROC MI and PROC MIANALYZE).

**Supplemental Figure 1.** Flowchart of the Study Design in the UK Biobank


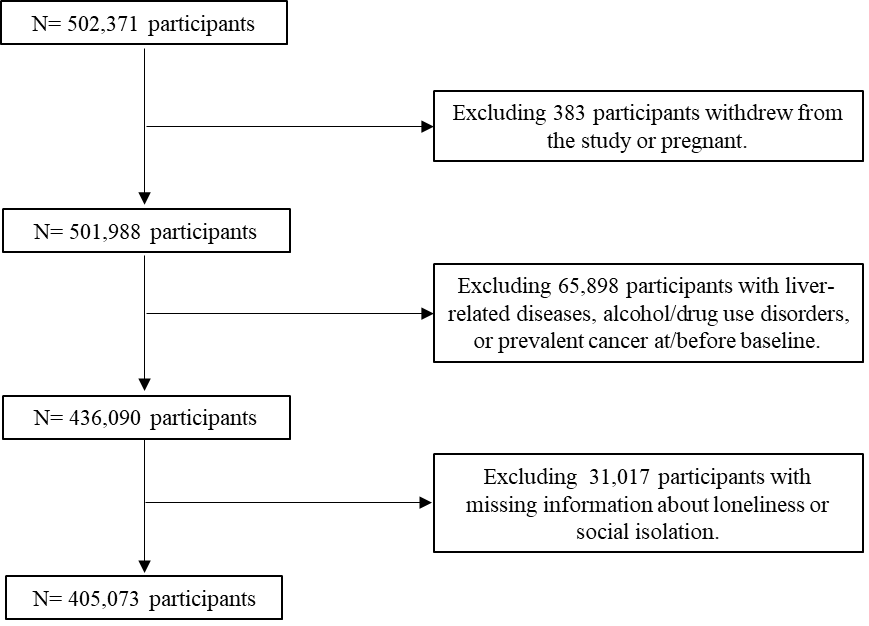

Supplement: Supplementary 1 — Fig. S1 Tables S1 to S9 [file hds.0220.f1.docx]
